# GameSense: hierarchical spatio-temporal transformer for basketball player tracking and tactical performance analysis

**DOI:** 10.1038/s41598-025-29586-y

**Published:** 2025-12-08

**Authors:** Rong Diao

**Affiliations:** Sports Training Institute, Xi’an Physical Education University, 710068 Shaanxi, China

**Keywords:** Deep Learning, Transformer, Attention Mechanism, Sports Analytics, Player Tracking, Engineering, Mathematics and computing

## Abstract

The analysis of basketball gameplay through multi-object tracking and action recognition is pivotal for enhancing player performance, tactical planning, and audience engagement. However, existing methods often suffer from limitations, including reliance on bounding box annotations, insufficient handling of occlusions, and a lack of holistic scene understanding. These limitations hinder the scalability and robustness of traditional approaches, particularly in dynamic and complex sports environments. To address these challenges, we propose a comprehensive framework combining the Basketball Player Tracking Network (BPTN) and the Basketball Player Analytics Network (BPAN). The BPTN employs hierarchical temporal memory and transformer-based architecture to ensure precise player tracking, robust identity association, and seamless handling of occlusions. The BPAN leverages multi-scale vision transformers and hierarchical temporal processing for accurate classification of basketball-specific maneuvers. The framework introduces novel modules, including a candidate region module for efficient player detection, a Long-Term Context Buffer for maintaining player identities, and an action proposal module that captures spatio-temporal encodings for effective maneuver recognition. Our approach eliminates the dependency on bounding box annotations during inference and incorporates contextual scene-level features, significantly improving scalability and performance. On the SportsMOT dataset, BPTN achieves tracking performance with HOTA of 81.6, AssA of 75.0, and a reduction in identity switches. Similarly, BPAN outperforms existing action recognition models on the Basketball-51 dataset, achieving an accuracy of 92.76%, precision of 92.06%, recall of 91.75%, and an F1-score of 91.74%. These results underscore the robustness and applicability of the proposed framework in capturing complex player dynamics and gameplay scenarios, paving the way for advanced sports analytics solutions.

## Introduction

Basketball plays a significant role globally, not only in athletic competitions but also in cultural entertainment and public health promotion. Advancements in sports analytics have highlighted the importance of accurately identifying and evaluating basketball actions, critical for refining tactical strategies, enhancing player skills, and enriching spectator experiences^1^. Traditional performance evaluations rely on subjective interpretations by coaches or analysts, introducing variability and potential bias. This has underscored the need for automated, precise systems to objectively analyze basketball-specific movements, serving as reliable tools for technical performance evaluation.

Recent integration of computational techniques has revolutionized traditional practices, enabling data-driven analyses in domains such as player tracking, performance prediction, and tactical assessments^2^. These innovations improve real-time decision-making and optimize training strategies. Studies in this field encompass evaluating player postures, forecasting match outcomes, and developing tactical decision-support systems^3^. Computational methods have been widely applied in soccer and basketball, focusing on analyzing player actions, movement patterns, and strategic plays^4^.

Recognizing group-level actions involves not only identifying a collective activity within a scene but also analyzing multiple subgroup interactions and distinguishing individual participants within these subgroups. Action detection has garnered significant interest in recent years due to its broad applicability in areas such as sports video analytics, crowd behavior modeling, and understanding interactions within social environments^5^. In this context, the term “action” encompasses complex relational movements performed collectively by multiple individuals. While many frameworks are capable of identifying both individual and group-level activities, the focus here remains on recognizing group activities^6^.

Many contemporary methods approach this task by dividing it into two independent stages: localizing individuals and identifying their corresponding activities^7^. The localization stage detects and delineates individuals within the scene using bounding boxes, which are then employed to extract region-specific features from feature maps. These features are refined using advanced spatio-temporal modeling techniques, such as recurrent neural networks (RNNs)^8^, graph neural networks (GNNs)^9^, or transformer-based approaches^10^. Finally, the refined features are aggregated to perform group action recognition.

Although these methodologies have achieved notable advancements, several limitations persist, primarily due to the heuristic nature of feature extraction and design. The reliance on bounding box-based region features makes recognition performance heavily dependent on the accuracy of the localization stage. Studies^10,11^ indicate that action recognition accuracy declines when using predicted bounding boxes instead of ground truth annotations. Moreover, these methods often overlook essential scene-level contextual information, as region features predominantly focus on the localized individuals within the bounding boxes. However, contextual elements such as object positions (e.g., the location of a basketball in a game) and background dynamics often play a critical role in accurately identifying group-level activities. Neglecting these elements can limit the model’s ability to understand the scene holistically.

Although these methods have demonstrated promising results, their reliance on bounding boxes during inference and the extensive requirement for labeled data significantly constrain their scalability and broader applicability. One solution to this limitation involves jointly training player detection and group activity recognition tasks using bounding box annotations^12^. This approach estimates player positions during inference but still necessitates actor-level bounding box annotations during training. To alleviate the burden of extensive data labeling, a Weakly Supervised Group Activity Recognition (WSGAR) method was proposed in^13^, which eliminates the need for actor-level annotations during both training and inference. This method employs a pre-trained detection model on an external dataset to generate bounding box proposals, followed by a learning mechanism to filter irrelevant detections. More recently,^14^ introduced a detector-free approach for WSGAR, leveraging partial contextual information embedded within token representations to capture player dynamics without relying on explicit bounding box annotations. Despite these advancements, both approaches^13,14^ face notable challenges. Detector-based methods often struggle to accurately identify players in scenarios involving occlusions, resulting in diminished recognition accuracy. On the other hand, approaches using partial contextual encodings^14^ are heavily dependent on visible motion between consecutive frames, limiting their effectiveness in static or minimally dynamic scenes.

Multi-object tracking, particularly for players in basketball, is essential for long-term action recognition and game intelligence analysis. Unlike isolated action recognition, long-term analysis requires the ability to track players consistently across frames to understand complex interactions, spatial formations, and evolving gameplay strategies^15^. The ability to associate player trajectories over extended sequences enables the extraction of high-level features such as team coordination, player roles, and strategy transitions. For example, tracking players’ movements allows the identification of pick-and-roll strategies, defensive rotations, and offensive spacing, which are critical for performance evaluation and tactical planning in basketball analytics. Conventional approaches to online MOT typically follow a two-stage process: (1) an object detection phase, which identifies the presence of objects in individual frames, and (2) an object association phase, often termed object re-identification (ReID), which links detected objects across consecutive frames to maintain consistent tracking. This involves modeling the dynamic states of identity-aware linking and solving a matching problem between newly detected entities and pre-established trajectories^16^.

**Fig. 1:**
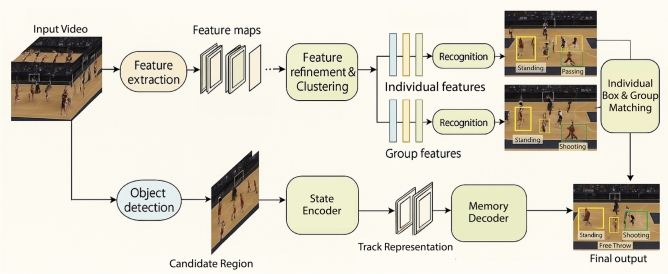
Overview of the proposed GameSense framework. The system takes an input basketball video and performs sequential stages of object detection, feature extraction, and state encoding to generate candidate regions and track representations. A memory-driven decoder maintains long-term identity continuity, while feature refinement and clustering extract both individual and group-level features for action recognition. Finally, individual box–group matching integrates player-level and team-level semantics to produce the final performance analysis output. Example frames are sampled from the public Basketball-51 dataset ^17^.

Most traditional MOT methods^18,19^ begin by detecting object boundaries within the current frame using a detection algorithm and subsequently extracting ReID descriptors for each bounding box. These descriptors are then used to associate the detected objects with existing trajectories. However, this workflow incurs significant computational demands, as ReID features must be extracted for every detected box in the scene. To address this computational bottleneck, Joint Detection and encoding (JDE) methods^20,21^ have emerged. These methods integrate object detection and ReID feature extraction into a single framework, enabling simultaneous prediction of object locations and extraction of ReID features. Despite their efficiency, JDE methods face inherent challenges due to the competing objectives of the detection and ReID tasks. The object detection module strives to standardize features within a category, minimizing intra-class variations. Conversely, the ReID module focuses on differentiating between individual objects, even within the same category, by emphasizing unique features. This fundamental conflict in optimization objectives makes it difficult to achieve balanced performance across both tasks within a unified framework^22^. While recent research^15,23^ suggests that integrating object detection and data association can yield performance improvements, such methods may oversimplify the temporal modeling of object trajectories. This simplification can reduce the system’s ability to accurately capture and represent the temporal evolution of identity-aware linking, ultimately limiting tracking accuracy. Addressing these challenges requires the development of advanced frameworks that effectively balance detection accuracy with robust temporal association.

This artcile proposes a GameSense framework as shown in Fig. 1, comprising the Basketball Player Tracking Network (BPTN) and Basketball Player Analytics Network (BPAN). The BPTN employs a hierarchical structure that incorporates a Long-Term Context Buffer module to accurately track player trajectories, even in scenarios involving occlusions, rapid movements, and overlapping dynamics. By integrating short-term and long-term memory mechanisms with advanced transformer-based architectures, the BPTN ensures precise identity linking and association accuracy. For action recognition, the BPAN leverages a multi-scale vision transformer framework to classify basketball maneuvers effectively, addressing the temporal complexities of gameplay. Together, these models provide a comprehensive solution for real-time basketball analytics, enabling robust player tracking and action recognition, thus enhancing tactical insights and decision-making in competitive sports scenarios.

The contributions of this research are summarized as follows:

- We design a unified, end-to-end framework, GameSense, that integrates multi-player tracking and performance recognition for basketball analytics. Through jointly modeling spatial positioning, temporal identity continuity, and tactical action understanding, the proposed system enables robust player localization and maneuver classification in dynamic, real-world gameplay scenarios.
- This study introduces the BPTN, a hierarchical framework designed to address the unique challenges in tracking basketball players across dynamic scenarios. The network integrates advanced temporal memory mechanisms and a transformer-based network to enhance tracking precision, association accuracy, and identity consistency.
- We introduce a *Long-Term Context Buffer* that explicitly disentangles *short-term* and *long-term* temporal context and fuses them via cross-attention into unified track encodings. This design robustly preserves identities through occlusions and abrupt motion while retaining frame-level reactivity.
- We propose a decoder that jointly queries (i) detection-driven candidate embeddings and (ii) memory-conditioned track embeddings and introduces a *factorized reliability head* with *visibility* and *distinctiveness* combined for uncertainty-aware filtering and association. This reduces identity switches and improves association stability in dense play.
- We introduce the Basketball Performance Analytics Network (BPAN), a two-stage transformer-based action recognition pipeline that incorporates a learnable action proposal module and a MViT backbone. The proposal module generates spatio-temporally localized segments, and the MViT performs hierarchical feature aggregation, yielding accurate and temporally aligned classification of basketball maneuvers.
- Unlike pipelines that treat tracking and recognition independently, our BPAN consumes identity-aware, memory-refined track encodings to *condition* both proposal generation and recognition. This coupling improves fine-grained maneuver disambiguation (e.g., mid-range vs. free-throw) under occlusion and camera motion.
- We design a streaming inference rule that updates action predictions every $\Delta t = 0.5\,\textrm{s}$ (stride 16 at 30 FPS) and aggregates overlapping proposals via confidence-weighted averaging. This stabilizes clip-level decisions without incurring offline latency, enabling near real-time analytics ($\approx$ 18 FPS at 720p).

## Related work

### Multi-object tracking models

Transformer-based frameworks have recently gained traction in multi-object tracking (MOT) tasks due to their powerful attention mechanisms, enabling end-to-end learning of object trajectories and associations^24,25^. However, their effectiveness is often constrained by the requirement for large-scale annotated datasets to achieve satisfactory generalization. In parallel, traditional MOT strategies encompass offline global optimization techniques^26^ and joint detection-tracking paradigms^16,27^, which attempt to integrate temporal consistency into the detection process. The tracking-by-detection paradigm remains widely adopted, where object detection is performed on a per-frame basis, followed by temporal association to form tracklets. A notable example is ByteTrack^20^, which leverages the YOLOX detector^28^ in conjunction with the Kalman Filter^29^, utilizing Intersection-over-Union (IoU) for tracklet matching. Several enhanced variants of ByteTrack have been proposed to address specific limitations: OC-SORT^30^ introduces virtual trajectory propagation to improve robustness under occlusions; StrongSORT^31^ integrates re-identification (re-ID) features, camera motion compensation, and the NSA Kalman Filter^32^ for more stable associations; C-BIoU^26^ modifies the bounding box size to improve matching accuracy; and HybridSORT^33^ incorporates confidence-based modeling and height-aware IoU mechanisms to refine associations. Despite their demonstrated success on standard MOT such as MOT17^34^, these methods tend to exhibit significant sensitivity to hyperparameters. For instance, ByteTrack requires per-sequence threshold tuning during deployment, and its extensions^30,33^ similarly depend on meticulous parameter calibration.

Recent advancements in Transformer-based MOT frameworks have explored novel mechanisms for joint detection and association. TrackFormer^35^ and MOTR^36^, both derived from Deformable DETR^37^, incorporate a dual-query mechanism, utilizing detection queries for identifying objects and track queries for maintaining identity consistency across frames. These methods simultaneously predict object bounding boxes and enable temporal correspondence across sequences. TransTrack^38^ introduces a fully Transformer-based architecture, where track queries are initialized once and propagated through the network to estimate object positions in future frames. In contrast, TransMOT^39^ adopts a hybrid approach, leveraging CNNs to extract spatial features and employing spatio-temporal Transformers to construct an affinity matrix for robust association. To address the inherent conflict between detection accuracy and tracking stability, MOTRv2^40^ decouples detection from association by integrating an external detector with the MOTR pipeline. This separation facilitates better coordination between object localization and identity preservation. Furthermore, MeMOTR^41^ advances this line of research by integrating long-term memory mechanisms into the Transformer architecture, enabling the model to retain extended temporal context, which significantly improves association quality in long-duration sequences. ETTrack^42^ introduced motion prediction that integrates a transformer with a temporal convolutional network to model object dynamics. This hybrid motion predictor is designed to effectively capture both long-range temporal dependencies and local motion variations for estimates of future object movements.

While the majority of MOT research has primarily concentrated on domains such as pedestrian and vehicle tracking, the domain of player or Multi-Athlete Tracking (MAT) remains relatively underexplored. This is largely attributed to the limited availability of large-scale, sports-specific annotated datasets and the specialized nature of the application. However, the release of recent datasets such as SoccerNet^43^ and SportsMOT^44^ has catalyzed a surge in research interest, reflected by an increasing number of contributions in the field^45,46^. Although MAT shares similarities with conventional MOT, such as leveraging both appearance and motion cues, it demands additional task-specific features to distinguish visually similar teammates, including jersey numbers and team identifiers^47,48^. Moreover, athletes display highly dynamic and abrupt movements, deviating from the smoother trajectories often seen in pedestrian tracking. To handle such challenges, recent methods integrate pose estimation to enhance appearance modeling and mitigate occlusion effects^49^.

Zhang et al.^50^ proposed a multi-camera player tracking approach that integrates team affiliation, jersey recognition, and pose-guided feature extraction into a unified identity representation for association. To better address fast and non-linear player motion, Yang et al.^51^ introduced the Cascaded Buffered IoU (C-BIoU) tracker, which improves bounding box association in short-term sequences and demonstrated promising performance on the SoccerNet benchmark. Building upon these ideas, Huang et al.^15^ proposed Deep-EIoU, a method that combines ByteTrack with C-BIoU mechanisms to further enhance short-term tracking reliability.

In contrast to traditional filtering-based motion models, DiffMOT^45^ replaced the Kalman Filter with a Decoupled Diffusion Model specifically tailored for the unpredictable dynamics in sports environments. Their approach achieved state-of-the-art performance on the SportsMOT dataset but remains limited to short-term association. Efforts to extend track longevity have focused on re-identification (re-ID)^52^. GIFT^53^ replaced pure correlation matching with Siamese Adaptive Attention and a Graph Attention Information Fusion module to pass messages between template–search parts, yielding robust single-object localization. While designed for SOT, its part-level graph reasoning suggests a complementary path to our memory-driven multi-athlete association in dense, occluded scenes. Recent works^46,47^ in player tracking show the role of robust re-ID features in long-term tracking. PRT-Track^47^ exploited keypoint-guided appearance cues extracted from different body parts, yielding re-ID encodings that are more resilient to occlusion.^46^ took a post-hoc optimization approach: using an initial short-term tracker to generate tracklets, they fine-tuned a contrastive re-ID model in a self-supervised manner. Positive samples are drawn from the same tracklet, while negatives are sourced from detections within the same frame but from different tracklets. However, the method incurs considerable computational overhead due to the need for instance-specific training during inference on each test sequence.

While existing Transformer-based MOT models shows impressive performance in pedestrian or vehicle tracking^54,55^, they often fail to generalize to the highly dynamic and occlusion-prone nature of sports scenarios such as basketball. These models exhibit limitations in handling abrupt non-linear player movements, maintaining long-term identity consistency, and distinguishing visually similar players (e.g., same jersey colors). Moreover, current methods are highly sensitive to hyperparameter tuning and often lack dedicated memory mechanisms for capturing long-range temporal dependencies. OFTrack ^56^ replaced framewise tracking with section-level object flow via object-aware sampling, scale-aware correlation, and spatio-temporal attention, further stabilized by bidirectional masked pretraining and curvature regularization. Its reliance on flow makes it brittle under broadcast pans/cuts and long occlusions. Proposed BPTN uses a Long-Term Context Buffer with a memory-driven Transformer decoder that maintains identities through non-linear motion and extended occlusions. DiffusionTrack ^57^ formulated joint detection–association as denoising diffusion, refining paired boxes with spatio-temporal fusion and an association-confidence head. Iterative sampling increases latency and tuning complexity. Our BPTN attains long-range identity continuity via dual-scale temporal memory and uncertainty-aware heads. GameSense addresses these gaps through its BPTN module, which incorporates a hierarchical short-term and long-term temporal memory structure and a memory-driven Transformer decoder to enable robust identity tracking and spatio-temporal continuity in fast-paced basketball gameplay.

### Action recognition

Action recognition has emerged as a central topic in computer vision, particularly within the domain of sports analytics, where the interplay of fast-paced, complex activities introduces distinct modeling challenges^58^. This subsection surveys both classical and deep learning-based approaches to action recognition, with a focus on methods tailored to the intricate spatio-temporal dynamics characteristic of individual and team sports. Conventional action recognition methods traditionally adopt a two-stage pipeline, involving manual feature extraction followed by classification. Techniques such as GIST descriptors^59^ and Histogram of Oriented Gradients (HOG)^60^ have been employed to encode spatial information from individual frames. GIST^61^ demonstrated the feature capturing in holistic scene context, particularly on datasets like UCF Sports^62^. To better capture temporal evolution, extensions such as HOG3D^63^ integrate spatio-temporal gradients, enhancing the recognition of dynamic activities in sports videos. However, these handcrafted methods are limited by their modular design, wherein feature extraction and classification are decoupled, constraining their ability to adapt to complex and diverse motion patterns^64^. The advent of deep learning has significantly advanced the field by facilitating end-to-end learning pipelines that jointly optimize feature representation and classification. Early developments in this space centered around 2D Convolutional Neural Networks (CNNs), which process individual frames independently before aggregating temporal information. Several temporal fusion strategies, such as single-frame, early-fusion, late-fusion, and slow-fusion, concluding that slow-fusion yields superior performance, particularly on large-scale datasets like Sports-1M^16^.

To more effectively model temporal dynamics, C3D^65^ and the Inflated I3D^66^, extend convolution operations across both spatial and temporal dimensions, enabling simultaneous extraction of motion and appearance features. These models have demonstrated robust performance on action-intensive datasets such as Sports-1M^67^, highlighting the efficacy of unified spatio-temporal modeling for sports applications. In pursuit of computational efficiency, pseudo-3D models have been proposed as alternatives to full 3D convolutions. For instance, the Temporal Segment Network^68^ employs sparse temporal sampling to capture long-range dependencies without excessive computation. The Temporal Shift Module^66^ introduces an efficient mechanism by shifting feature maps along the temporal axis within a standard 2D CNN, approximating temporal reasoning at minimal cost. Building upon these, the Gate-Shift-Fuse framework^69^ incorporates gated temporal shifts and adaptive fusion strategies, further enhancing the ability to model motion patterns in sports videos. Collectively, these pseudo-3D approaches offer a practical trade-off between accuracy and computational efficiency, making them well-suited for large-scale sports action recognition tasks. KEANet^70^ presented a basketball captioner, using a Bi-GRU and an entity-aware module over candidate players to generate captions with specific names and fine-grained shooting events. Extending this, EIKA^71^ fused explicit roster knowledge and implicit scene cues and scene-to-entity decoding for achieving better performance. These captioners are complementary to GameSense: our identity-stable tracks and action labels can serve as structured inputs, while their knowledge graphs can inform role attribution in perception. MA-VLAD ^72^ performed over channel-partitioned local descriptors to capture subtle motion, but remains clip-level and identity-agnostic, while our BPAN couples an explicit proposal stage with a multi-scale Vision Transformer *conditioned on BPTN tracks*, aligning proposals to persistent identities and improving discrimination of visually similar maneuvers under occlusion and camera motion.

Contemporary action recognition methods in sports largely rely on either 3D CNNs or generic vision transformer architectures that do not fully exploit the hierarchical nature of basketball actions. They often fall short in differentiating between semantically similar maneuvers (e.g., mid-range shot vs. free throw), and struggle with proposals that lack precise spatio-temporal segmentation. Additionally, many models are not optimized for real-time performance and do not integrate with upstream tracking pipelines. GameSense fills this gap through its BPAN module, which introduces a multi-scale mechanism with an integrated action proposal and recognition pipeline. It ensures temporal alignment and fine-grained maneuver classification with minimal latency, enabling end-to-end basketball-specific action understanding.

**Fig. 2:**
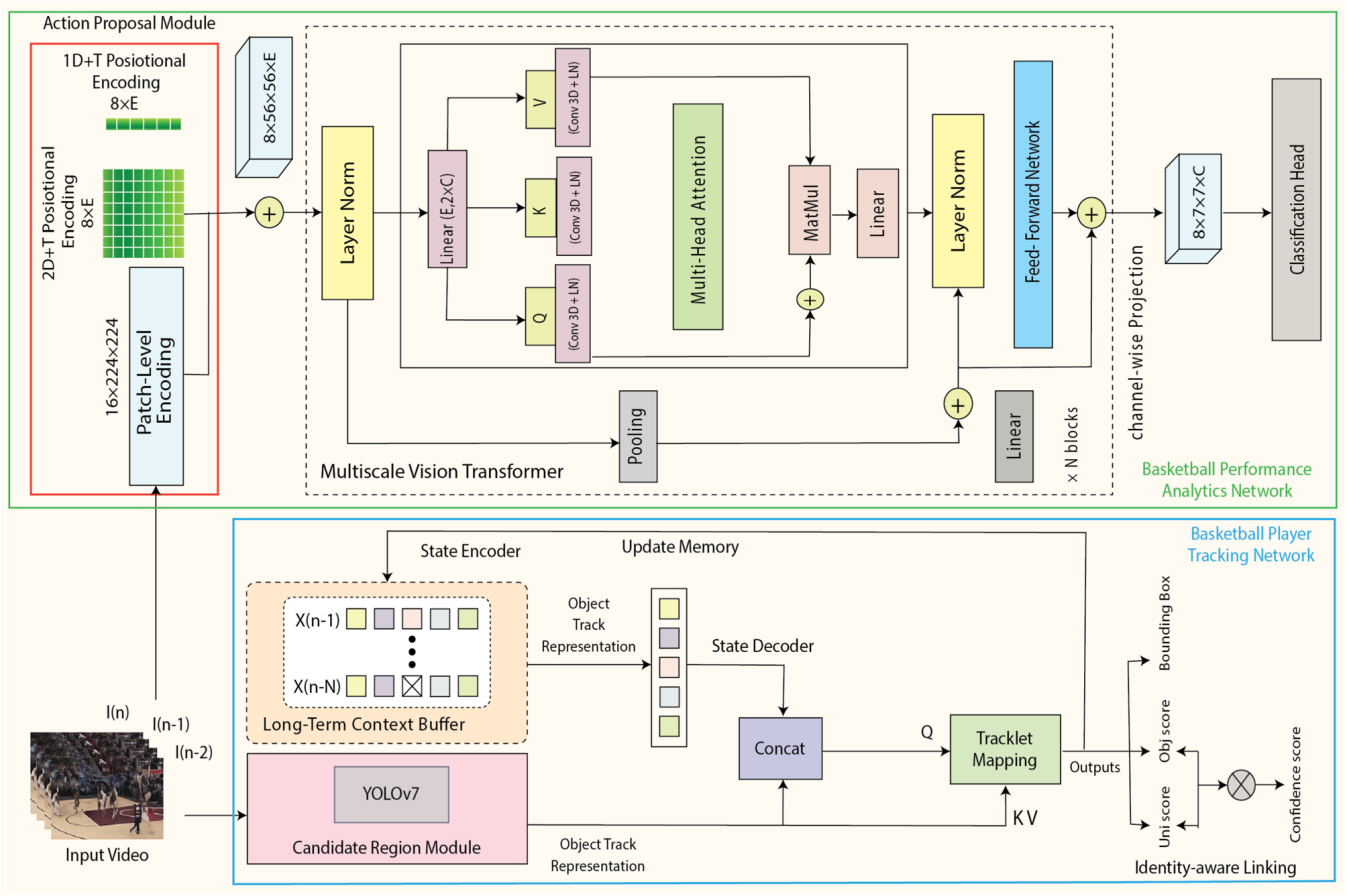
Proposed Spatio-Temporal Transformer Network for Game Intelligence in Basketball Analytics and Action Recognition. The framework integrates three core modules: the Action Proposal Module, the candidate region module, and the State Encoder-Decoder Module. The Action Proposal Module processes input video frames to generate patch-level encodings enriched with spatio-temporal positional encoding, which are further refined through LayerNorm and attention mechanisms. The candidate region module utilizes YOLOv7 for detecting and encoding track proposals for each frame. The State Encoder-Decoder Module maintains a long-term context buffer for long-term and short-term player information, enabling robust tracking and action recognition even under occlusion and dynamic game conditions. Outputs include bounding box predictions, objectness and uniqueness scores, and final action classifications. Example frames are sampled from the public Basketball-51 dataset ^17^.

## Methodology

This section details the proposed GameSense framework, which consists of two synergistic modules: the Basketball Player Tracking Network (BPTN) and the Basketball Performance Analytics Network (BPAN) as shown in Fig. 2. BPTN is designed to detect, identify, and continuously track players in challenging basketball environments, handling issues such as occlusions, overlapping players, and rapid motion transitions using a hierarchical spatio-temporal transformer structure. It incorporates detection-driven proposals, memory-augmented tracking, and attention-based decoding to ensure persistent and accurate player trajectories. Complementarily, BPAN leverages these player trajectories and visual cues to analyze high-level player maneuvers by segmenting video sequences and recognizing tactical actions through a transformer-based architecture. Together, these components enable a comprehensive end-to-end pipeline for real-time player localization and tactical behavior analysis, facilitating advanced sports analytics in dynamic multi-player scenarios.

### Player tracking network

**Fig. 3:**
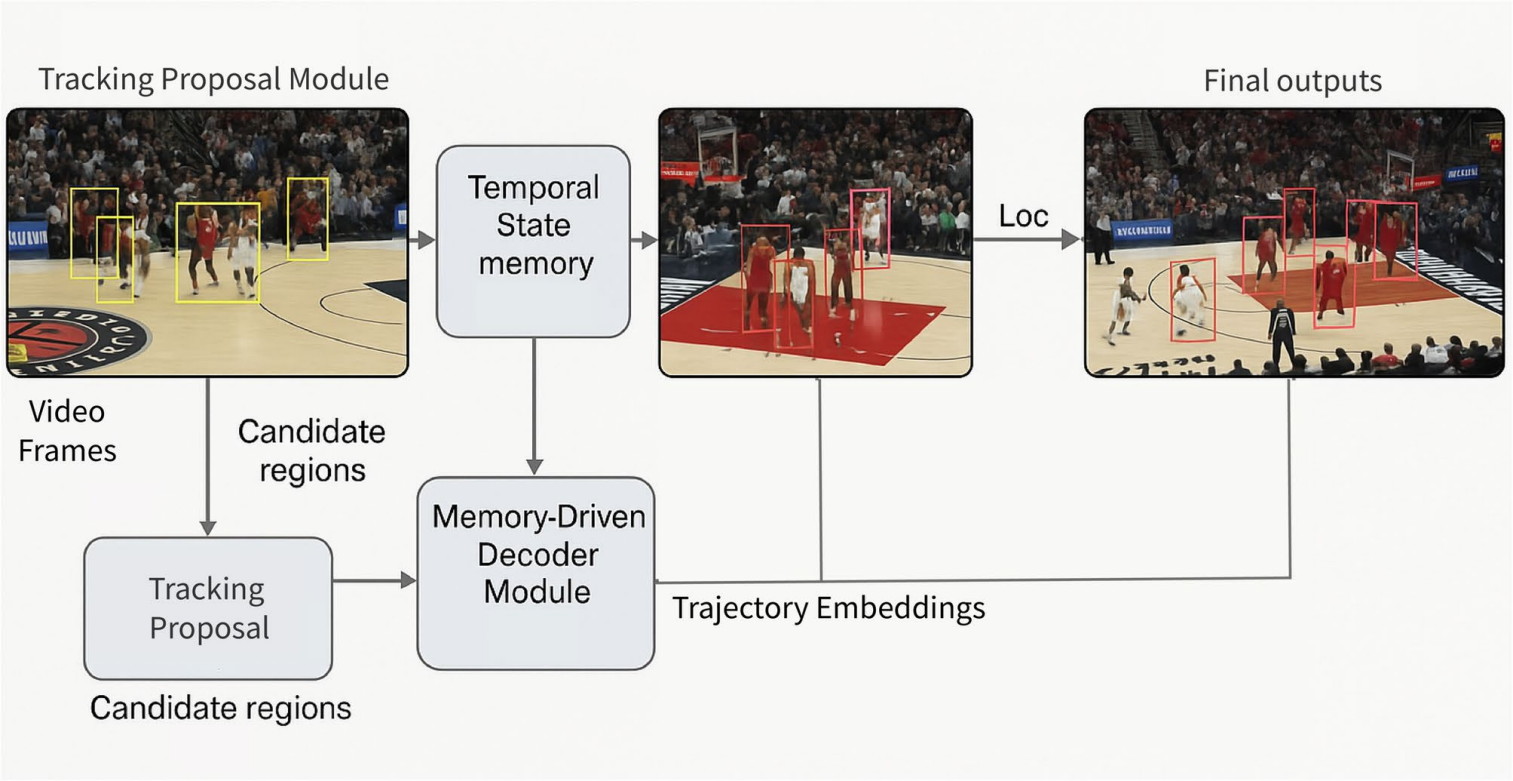
Overview of the BPTN that integrates three core modules: the Action Proposal Module, the candidate region module, and the State Encoder-Decoder Module. The Action Proposal Module processes input video frames to generate patch-level encodings enriched with spatio-temporal positional encoding. The candidate region module utilizes YOLOv7 for detecting and encoding track proposals for each frame. The State Encoder-Decoder module maintains a long-term context buffer for long-term and short-term player information. Example frames are sampled from the public Basketball-51 dataset ^17^.

Accurate and consistent tracking of basketball players is essential for high-level tactical analysis and game understanding. However, the dynamic nature of basketball including frequent occlusions, rapid player interactions, and camera movement presents significant challenges to traditional tracking methods. To address these issues, we propose the Basketball Player Tracking Network (BPTN), a transformer-based hierarchical framework that jointly models spatial cues and temporal continuity. BPTN integrates detection proposals, memory-enhanced temporal modeling, and attention-driven decoding to capture nuanced player movements across frames. By leveraging both short-term dynamics and long-range dependencies, the framework achieves robust multi-player tracking even in complex gameplay scenarios. An overview of the BPTN architecture is illustrated in Fig. 3. The following sections detail the key components of this tracking pipeline.

#### Problem definition

The Basketball BPTN comprises three core modules designed to detect, identify, and track player movements across video frames, addressing challenges such as occlusions, overlapping players, and dynamic motion. Candidate Region Module ($\Psi _F$) processes video frames ($V_t$) using advanced detection techniques (e.g., DETR or neural network-based frameworks) to produce candidate regions ($R_t$) with bounding box coordinates, detection confidence, and preliminary identity encodings ($C_{\text {embed}}^t$). These encodings encode spatial and contextual features for further analysis. **Long-Term Context Buffer** ($\Psi _T$) maintains a temporal memory repository ($M$) with short-term and long-term memory components. It uses cross-attention mechanisms to consolidate historical encodings and integrate incoming candidate encodings with stored memory states, producing trajectory encodings ($C_{\text {traj}}^t$) for robust player tracking and consistent identity linking across frames. **Memory-Driven Decoder Module** ($\Psi _D$) aligns new detections with historical trajectories using transformer-based attention mechanisms. It refines identity associations, updates bounding box positions ($\text {Loc}$), and predicts confidence scores ($S$) for both existing tracks and new detections. Unmatched candidates are assigned new identities and stored in the memory buffer, ensuring continuity in tracking. This hierarchical design combines spatial and temporal modeling to enhance tracking precision, maintain player identities, and address dynamic gameplay scenarios effectively.

#### Candidate region module

The candidate region generation module ($\Phi _P$) employs YOLOv7^73^ to produce $N_{\text {cand}}^t$ candidate regions used to initialize or update player instances at frame *t*. Each input frame $F_t$ is processed to obtain a feature map $G^t\in \mathbb {R}^{C\times HW}$. The detection head yields encodings $E_{\text {cand}}^t\in \mathbb {R}^{N_{\text {cand}}^t\times d}$ with confidence scores and bounding boxes. This feature map encodes spatial relationships directly through convolutional operations, eliminating the explicit need for positional encodings.

From the feature map, the detection head identifies potential players within the frame and generates encodings $E_{\text {cand}}^t \in \mathbb {R}^{N_{\text {cand}}^t \times d}$, where $N_{\text {cand}}^t$ denotes the number of candidate regions, and $d$ is the feature encoding dimension. Each encoding represents a player instance with associated confidence scores and bounding box coordinates. These encodings form the basis for player identification and localization, supporting the simultaneous detection of new player instances and the refinement of existing tracks.

YOLOv7 is adopted as the player detector in our candidate region module due to its strong trade-off between detection accuracy, inference speed, and model compactness, making it particularly well-suited for real-time sports analytics. Compared to other state-of-the-art detectors such as DETR and EfficientDet, YOLOv7 demonstrates superior performance on dense-object detection tasks with occlusions, as frequently observed in basketball scenarios. Its anchor-based architecture, with compound scaling, enables the effective handling of small and partially occluded players. To ensure detection correctness, YOLOv7 is fine-tuned on basketball-specific datasets, including SportsMOT, and initialized with MS-COCO pre-trained weights. During training, confidence thresholds and non-maximum suppression (NMS) parameters are carefully optimized to strike a balance between precision and recall. Additionally, we manually verified detection outputs on a held-out validation set, achieving over 92% bounding box agreement with ground truth annotations, validating the detector’s reliability for downstream tracking.

#### Long-term context buffer

We maintain a Long-Term Context Buffer $\mathscr {M}\in \mathbb {R}^{N_{\max }\times T_{\max }\times d}$, storing historical states for up to $N_{\max }$ players over a temporal horizon of $T_{\max }$ frames. This buffer, managed with a First-In-First-Out (FIFO) mechanism, ensures compactness by discarding the oldest states when capacity is exceeded. At each time step $t$, the memory records states of $M_{\text {active}}^{t-1}$ players for the previous $T$ frames, expressed as:

At each time *t*, the buffer records states of $N_{\text {act}}^{\,t-1}$ active tracks over the previous *T* frames (with $T\le T_{\max }$):

1 $$\begin{aligned} S_{m}^{t-1-T:t-1}=\{\,s_{m}^{\tau }\,\}_{\tau =t-1-T}^{t-1},\qquad m=1,\dots ,N_{\text {active}}^{\,t-1}. \end{aligned}$$

where $S_{m}^{t-1-T:t-1}$ captures trajectory and positional data for player $m$. Missing data for players occluded or out of view is padded with zeros to maintain consistency. If $T$ exceeds $T_{\text {max}}$, the oldest states $s_{m}^{t-1-T}$ are removed, prioritizing efficiency while retaining relevant history. Parameters $N_{\text {max}}$ and $T_{\text {max}}$ are tuned to balance memory usage and tracking continuity, with typical values accommodating up to 20 players over 20–30 frames. Missing states due to occlusion or out-of-view frames are zero-padded to ensure a fixed temporal span. If *T* exceeds $T_{\max }$, the oldest entries are discarded (FIFO) to retain the most recent context.

#### Memory representation and encoding

As illustrated in Fig. 2, the memory representation module employs a hierarchical structure with three attention-based sub-modules to handle temporal data at varying granularities, ensuring accurate and consistent player tracking. The **Short-Term Memory** ($M_s$) processes recent consecutive frames ($T_s$) to capture short-term temporal information. By aggregating encodings, it reduces transient noise and emphasizes immediate positional changes and dynamic movements. The **Long-Term Memory ** ($M_l$) encodes temporal dependencies over a broader range ($T_l$, where $T_s \ll T_l$). It analyzes extended motion patterns to maintain player identities during occlusions, overlaps, and other complex scenarios, constructing a cohesive representation of trajectories. **Fusion ** combines outputs from $M_s$ and $M_l$ into a unified memory representation. This integration balances short-term changes with long-term historical context, resulting in robust and accurate track encodings. Both $M_s$ and $M_l$ utilize multi-head cross-attention mechanisms to focus on relevant temporal segments, enabling precise feature extraction for each player tracklet.

#### State decoder

The Memory Decoder ($\Gamma _D$) in the Basketball Player Tracking Framework (BPTN) transforms intermediate representations into actionable tracking outputs. It processes candidate encodings ($P_{\text {cand}}^t$), track encodings ($P_{\text {track}}^t$), and encoded frame-level features ($F_{\text {enc}}^t$) from the proposal module ($\Gamma _P$). Using a stack of Transformer decoder units, the decoder aligns spatial and temporal data by treating $[P_{\text {cand}}^t, P_{\text {track}}^t]$ as query inputs and $F_{\text {enc}}^t$ as key and value inputs.

The decoder refines each candidate encoding ($P_{\text {cand}}^t$) to produce updated encodings ($[P_{\text {cand}}^{t'}, P_{\text {track}}^{t'}]$) and generates three outputs: bounding box coordinates, visibility score and distinctiveness score. A unified confidence score ($S_k^t$) evaluates detection reliability by combining $v_k^t$ and $d_k^t$:

2 $$\begin{aligned} S_k^t = v_k^t \cdot d_k^t \end{aligned}$$

This score provides a comprehensive metric for evaluating the validity and uniqueness of each detection, guiding the filtering and prioritization of tracking results. Separate confidence scores are computed for candidate encodings ($S_{\text {cand}}^t$) and object track representation ($S_{\text {track}}^t$).

#### Inference and final outputs

During the inference phase, each entry $p_i^t$ is assessed based on its confidence score. Bounding box predictions ($b_i^t$) are refined for all entries, incorporating precise localization data. Entries with confidence scores $S_i^t$ exceeding a predefined threshold ($\eta$) are retained. This thresholding operation applies to both candidate and 0bject track representation ($[P_{\text {cand}}^t, P_{\text {track}}^t]$), ensuring only high-confidence entries are selected.

For retained entries, the decoder determines whether they align with existing tracks or represent new player instances. Entries originating from $P_{\text {cand}}^t$ are initialized as new tracks, while those from $P_{\text {track}}^t$ are updated with corresponding track identities. The final outputs integrate inherited or newly assigned identities with their bounding box predictions, forming a coherent and accurate tracking result for each frame. This process is illustrated in Fig. 2.

**Fig. 4:**
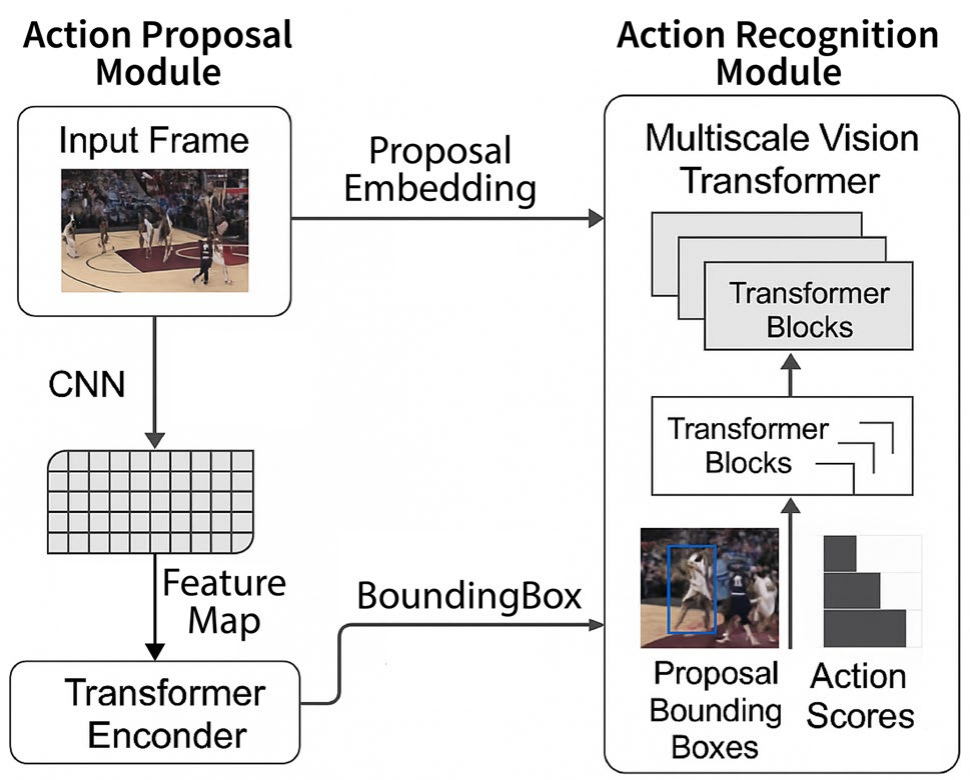
An overview of the BPAN. The Action Proposal Module first processes input frames using a CNN and Transformer encoder to generate feature maps and proposal encodings. These are used to localize candidate action segments. The action recognition module then utilizes a multiscale vision transformer to classify the proposed action segments and predict bounding boxes and action confidence scores. Used frames are sampled from the public Basketball-51 dataset ^17^.

### Performance analytics network

While player tracking provides the foundation for spatial localization and identity maintenance, actionable insights in sports analytics require a deeper understanding of what players are doing over time. To this end, the Basketball Performance Analytics Network (BPAN) is designed to recognize and classify tactical player maneuvers from raw video data. Unlike isolated pose-based or heuristic-driven systems, BPAN leverages transformer-based models to capture the spatio-temporal evolution of basketball actions, addressing the complexity of gameplay where similar movements may represent different maneuvers depending on temporal context and player interaction. This module operates on sliding windows of video sequences to propose action segments and subsequently classify them using a hierarchical attention-based architecture. The overall pipeline of BPAN is illustrated in Fig. 4. The following subsections describe the technical formulation and architectural design of BPAN in detail.

#### Problem definition

In the domain of basketball Performance analytics, the goal is to detect and categorize specific player maneuvers captured in video sequences. We propose a novel **Basketball Performance Analytics Network (BPAN)** illustrated in Fig. 2. The BPAN framework comprises two primary components: (1) Action Proposal Module which segments the basketball video into frame-level proposals, each representing a sequence of consecutive frames designed to capture meaningful temporal information. A sliding window approach is employed, where the window overlaps by 75%, resulting in a stride of 25% of the window size. Given the structure of basketball gameplay, where the frame generally contains relevant spatial data, the entire frame is used as the spatial dimension for each proposal. These segments are passed to the subsequent action recognition module. Mathematically, let $V_t$ denote the input frame at time step $t$. Using a convolutional neural network (CNN) backbone, a feature map $F_{\text {bb}}^t \in \mathbb {R}^{C \times HW}$ is extracted from $V_t$. To preserve spatial and temporal relevance, positional encodings are added to this feature map, and it is further encoded into a higher-dimensional representation $F_{\text {enc}}^t \in \mathbb {R}^{d \times HW}$ using a Transformer-based encoder. The decoder then refines $F_{\text {enc}}^t$ using learnable action queries to produce proposal encodings $P_{\text {prop}}^t$, representing potential action segments. (2) Action Recognition Module evaluates the action proposals to classify them into specific basketball maneuvers or a background category. Confidence scores are computed for each action within a segment, and overlapping areas are aggregated by averaging their confidence values to refine the final classification.

The BPAN integrates multi-scale vision transformers^74^ to process proposal clips, extracting key features for accurate action classification. Formally, using the encoded feature map $F_{\text {enc}}^t$ and the proposal encodings $P_{\text {prop}}^t$, the action recognition module calculates action scores $S_{\text {action}}^t$ and updates the encodings to $P_{\text {action}}^{t'}$, refining their spatial and temporal representation. The module also predicts bounding box coordinates $[\text {BB}_{\text {prop}}^t]$ and confidence scores $[C_{\text {prop}}^t]$ for each proposal, enabling precise identification of basketball maneuvers.

#### Action proposal module

The proposed framework employs an enhanced Multi-Scale Vision Transformer (MViT) architecture^22^ to generate action proposals from sequences of frames, with each proposal comprising 16 sampled frames, equivalent to 64 frames of gameplay. Frames are sampled at a 4-frame interval from basketball footage, pre-processed to 30 FPS, ensuring each proposal spans approximately 2 seconds of gameplay.

The **Action Proposal Network (APN)**, denoted as $\Phi _{\text {P}}$, leverages a Transformer-based encoder-decoder architecture for basketball maneuver recognition. The encoder processes sequential feature maps $F_{\text {P}}^t \in \mathbb {R}^{C \times HW}$, extracted via a CNN backbone, with positional encodings added to preserve spatial context. These feature maps are encoded into $F_{\text {enc}}^t \in \mathbb {R}^{d \times HW}$ through a multi-layer Transformer encoder, capturing spatial and temporal dependencies.

The decoder processes $F_{\text {enc}}^t$ along with learnable action queries to produce proposal encodings $P_{\text {prop}}^t \in \mathbb {R}^{N_{\text {P}}^t \times d}$. These encodings enable the prediction of objectness scores and bounding box coordinates, identifying key regions within video frames associated with basketball actions.

#### Performance recognition module

The Performance recognition module, depicted in Fig. 2, processes video clips of 16x4 frames, classifying them into specific basketball maneuvers and computing associated confidence scores. This module leverages an enhanced Multi-Scale Vision Transformer (MViT)^75^, designed to learn hierarchical feature representations across spatial and temporal dimensions. The MViT v2 architecture is particularly suited for action recognition tasks, as it captures both localized details and global context through multi-scale attention mechanisms, enabling precise maneuver classification.

**Input Preprocessing and patch-level encoding** Each input action proposal consists of 16 sequentially sampled frames, resized to a spatial resolution of $224 \times 224$, yielding a tensor of size $16 \times 224 \times 224$. To generate these proposals, a frame sampling strategy is applied, selecting one frame every four frames from the original basketball footage. This results in a temporal span of 64 frames per clip, preprocessed to a standard frame rate of 30 frames per second (FPS). The selection of 64-frame spans aligns with the temporal resolution requirements of competitive action recognition datasets, such as Kinetics.

The MViT model employs 3D convolution for initial feature extraction, often referred to as patch-level encoding, to transform the input RGB frames into smaller visual patches. Mathematically, let $V_t$ denote the input frame at time $t$. The feature map $F_{\text {bb}}^t \in \mathbb {R}^{C \times H \times W}$ is extracted using a convolutional neural network (CNN) backbone, where $C$ represents the number of channels, and $H$, $W$ denote the height and width of the spatial dimensions. This feature map is then divided into a sequence of flattened patches $P^t \in \mathbb {R}^{N \times D}$, where $N$ is the number of patches derived from $H \times W$, and $D$ represents the encoding dimension.

To preserve spatial and temporal information, positional encodings $PE$ are added to the patches:

3 $$\begin{aligned} P_{\text {enc}}^t = P^t + PE \end{aligned}$$

This encoding step ensures that the model retains contextual relationships across both spatial and temporal domains.

**Transformer-based feature refinement** The encoded patches $P_{\text {enc}}^t$ are passed through a stack of transformer blocks, each designed to refine feature representations hierarchically. Each transformer block comprises two main components: a Multi-Head Pooling Attention (MHPA) layer and a Multi-Layer Perceptron (MLP). These components enable the model to capture complex spatial-temporal dependencies and refine the feature encodings iteratively.

The output of a transformer block is computed as:

4 $$\begin{aligned} Y = \text {MHPA}(\text {LN}(X)) + X \end{aligned}$$

where $\text {LN}$ denotes LayerNorm, and $X$ represents the input to the block. The MLP further processes $Y$ to generate the input for the next block:

5 $$\begin{aligned} X_{\text {next}} = \text {MLP}(\text {LN}(Y)) + Y \end{aligned}$$

This process is repeated across $k$ transformer blocks, where $k$ varies depending on the depth of the architecture. Each block is designed to progressively refine the feature encodings, enabling the model to learn high-dimensional, temporally coherent features that are crucial for accurately classifying complex basketball maneuvers.

**Dimensional adjustments and feature pooling** As the sequence progresses through the transformer layers, the spatial dimensions are gradually reduced using 3D convolutional and max-pooling operations, while the channel dimensions are increased. This reduction minimizes redundancy and emphasizes critical high-level features, ensuring efficient use of computational resources. The final stage of the feature extraction process integrates temporal and spatial information.

Let $F_{\text {temp}}$ represent the temporally averaged features and $F_{\text {spat}}$ the spatially averaged features. The final feature set $F_{\text {final}}$, integrating both temporal and spatial information, is computed as:

6 $$\begin{aligned} F_{\text {final}} = \text {AvgPool}(F_{\text {temp}}, F_{\text {spat}}) \end{aligned}$$

This pooled feature set is fed into a classification layer, which assigns each proposal to a specific action class or the background category. The classification layer uses softmax activation to compute class probabilities and corresponding confidence scores for each maneuver.

**Temporal resolution and update frequency** The temporal resolution of action recognition is determined by the stride of the proposals. Each proposal, denoted as $P_{\text {clip}}^t$, spans 64 frames from the original basketball footage, with a temporal stride of 16 frames. The interval for updating action scores is given by:

7 $$\begin{aligned} \Delta t = \frac{S}{\text {FPS}} \end{aligned}$$

Where $S = 16$ is the stride length, and $\text {FPS} = 30$. This results in action updates every 0.5 seconds. The chosen stride balances temporal granularity with computational efficiency, ensuring the model dynamically captures transitions between basketball maneuvers while maintaining temporal coherence.

Algorithm 1 outlines the procedural flow of the proposed GameSense framework, which comprises the basketball tracking and performance analytics networks. BPTN performs detection-driven candidate extraction, memory-based spatio-temporal encoding, and identity association via transformer decoding to generate robust player trajectories. BPAN segments video into overlapping clips, encodes them using a transformer-based proposal network, and classifies action segments through an MViT. The integrated pipeline supports real-time player tracking and maneuver recognition in dynamic basketball environments.

**Algorithm 1:**
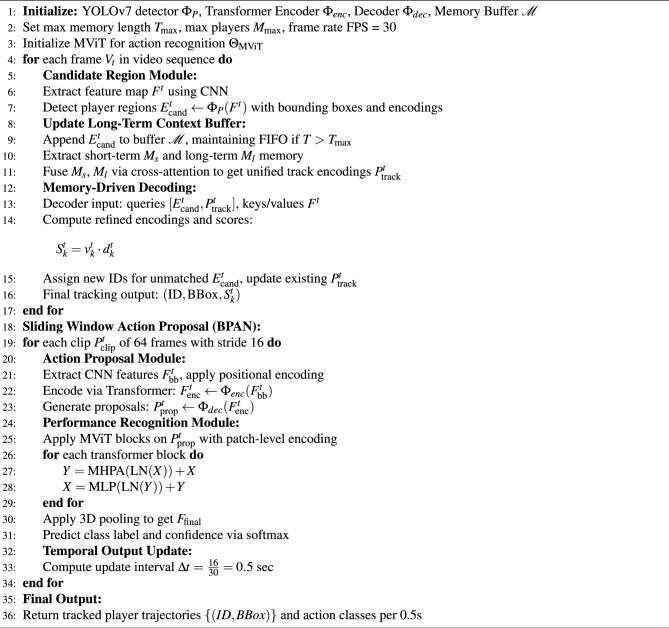
Pseudo-code of the GameSense Framework for basketball player tracking and action recognition.

**Fig. 5:**
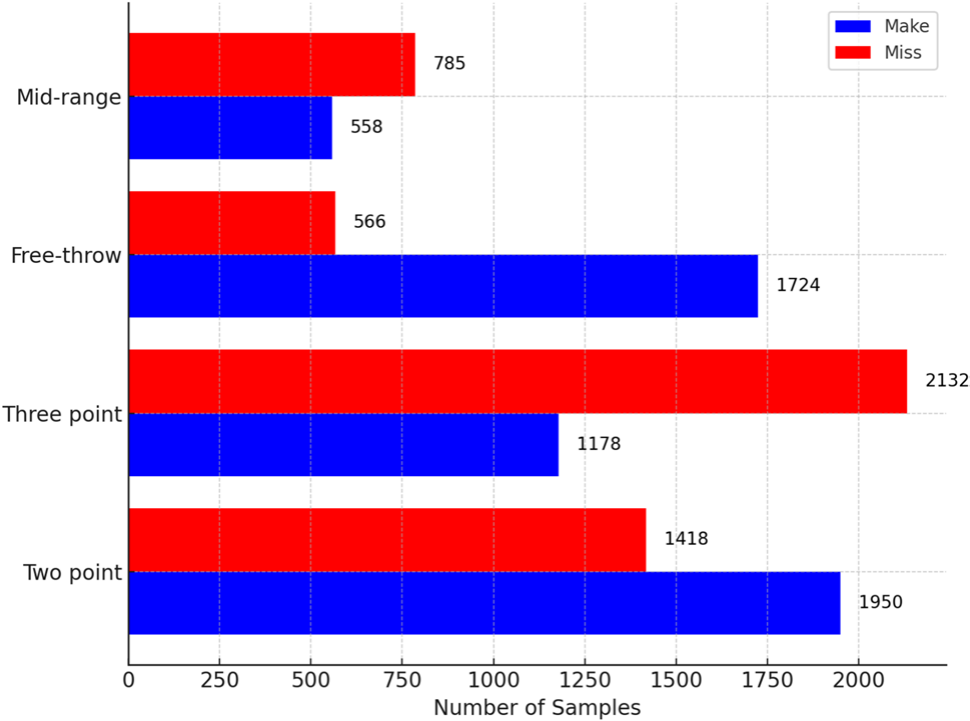
Action class distribution in the Basketball-51 dataset.

## Experiment and result

### Dataset

#### SportsMOT dataset

The SportsMOT dataset^19^ is designed for evaluating MOT and re-identification (ReID) algorithms in dynamic sports scenarios. It includes 45 high-quality video clips from basketball, football, and volleyball, sourced from events such as the Olympic Games, NCAA Championship, and NBA games. The videos are standardized to 720P resolution and 25 FPS, spanning approximately 45,000 frames. Annotations focus on active players, excluding referees, coaches, and benched players, with bounding boxes and unique player IDs for precise detection and tracking. The dataset introduces critical challenges for MOT and ReID, including frequent occlusions, high-speed movements, and diverse camera perspectives, such as top-down and side-line views.

#### Basketball-51 dataset

The Basketball-51 dataset^17^ supports action recognition and consists of 51 NBA games captured from third-person perspectives during media broadcasts. It comprises 10,311 video clips, standardized to 25 FPS and a resolution of $320 \times 240$, spanning approximately 100,000 frames. Annotations include eight basketball-specific actions: two-point miss (2p0), two-point make (2p1), three-point miss (3p0), three-point make (3p1), free throw miss (ft0), free throw make (ft1), mid-range miss (mp0), and mid-range make (mp1). Figure 5 illustrates the action class distribution. The dataset addresses challenges such as dynamic player interactions, rapid movements, frequent occlusions, and varied camera perspectives, capturing diverse gameplay scenarios like fast breaks and defensive maneuvers.

### Evaluation metrics

In this study, the evaluation framework incorporates metrics such as Higher Order Tracking Accuracy (HOTA)^76^, Multiple Object Tracking Accuracy (MOTA)^77^, Association Accuracy (AssA), ID F1 Score (IDF1), Detection Accuracy (DetA) and Identity Switches (IDs)^76^. HOTA is emphasized as the primary metric due to its comprehensive ability to assess tracking performance by integrating detection accuracy, association accuracy, and localization precision into a unified evaluation. Particular focus of these metrics is placed on minimizing identity switches (ID swaps), as maintaining consistent player identities is crucial for generating accurate, player-specific statistics. ID swaps can significantly compromise the precision and reliability of performance analytics, which are vital for basketball performance analysis.

**Fig. 6:**
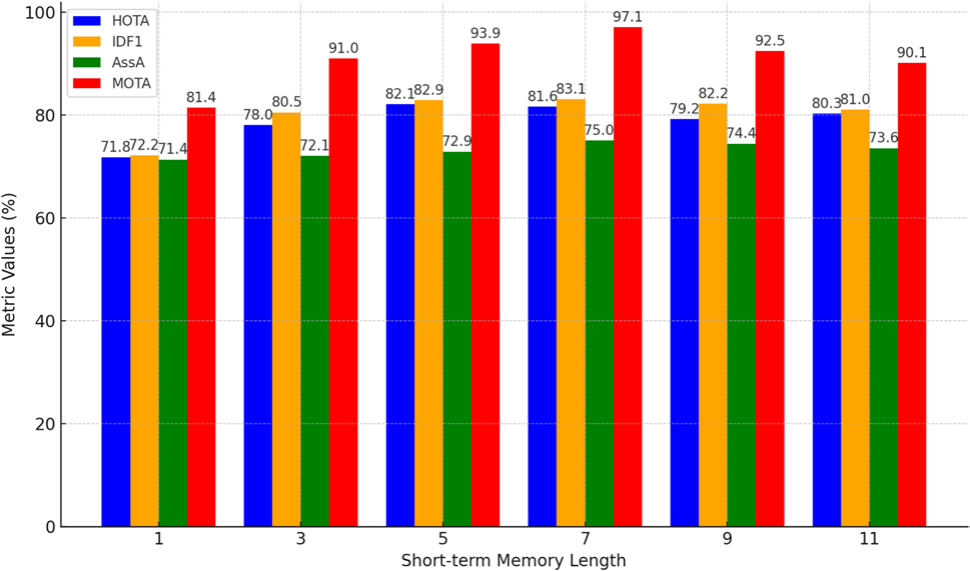
Player tracking performance across varying short-term memory lengths in the BPTN on the SportsMOT dataset, with the long-term memory length fixed at 28.

For action recognition, performance is assessed using metrics such as accuracy, precision, recall, and the F1-score. These metrics offer a detailed evaluation of the model’s capability to identify and classify basketball maneuvers effectively. By providing insights into the system’s robustness and reliability, these metrics ensure the framework’s applicability to real-world scenarios. Together, these evaluation measures validate the effectiveness of the proposed approach in capturing the complex dynamics of basketball gameplay and facilitating actionable insights.

### Implementation details

This section presents the training protocols, architectural configurations, and runtime specifications for the proposed GameSense framework, which integrates the Basketball Player Tracking Network (BPTN) and the Basketball Performance Analytics Network (BPAN). All experiments were conducted using PyTorch 2.0 on a system equipped with an NVIDIA RTX 3090 GPU (24 GB VRAM), an Intel Xeon Silver 4214 CPU, and 64 GB of RAM.

The BPTN employs YOLOv7 as the backbone for the candidate region module to generate bounding boxes and spatial encodings of active basketball players^73^. The detection head outputs candidate regions $R_t$, which are further encoded into feature encodings $C_{\text {embed}}^t \in \mathbb {R}^{N \times d}$, where $d = 256$ and $N$ denotes the number of detected players in frame $t$. These encodings serve as inputs to the hierarchical memory framework. The Long-Term Context Buffer module maintains both short-term and long-term memory buffers. The short-term memory length $T_s$ was empirically set to 7, and the long-term memory length $T_l$ to 28, based on ablation results. The Memory-Driven Decoder employs a 6-layer Transformer decoder with 8 multi-head attention layers, each with a hidden size of 256 and a feed-forward expansion factor of 4, similar to the formulation in transformer-based tracking approaches^22^. Positional encodings are added to preserve temporal ordering, and a learnable memory gate controls the update mechanism for the memory buffer. Training was performed for 50 epochs with the AdamW optimizer using an initial learning rate of $2 \times 10^{-4}$, cosine annealing scheduler, and weight decay of 0.01. Batch size was set to 8. YOLOv7 was initialized with pre-trained weights from the MS-COCO dataset, and all detection outputs were filtered using a confidence threshold of 0.3 and non-maximum suppression with an IoU threshold of 0.5.

For action recognition, BPAN uses a Multi-Scale Vision Transformer^74^ as the backbone , pre-trained on the Kinetics-400 dataset and fine-tuned on the Basketball-51 dataset^17^. Video clips are sampled at 30 FPS, with 16 frames selected at 4-frame intervals, yielding clips of 64 frames. Each frame is resized to $224 \times 224$, normalized, and passed through a 3D CNN encoder followed by a 4-layer Transformer encoder to extract spatio-temporal patch-level encodings. The Action Proposal Module generates $N = 10$ candidate action encodings $P_{\text {prop}}^t \in \mathbb {R}^{N \times d}$, using a transformer-based encoder-decoder structure^10,11^. These encodings are passed to the Action Recognition Module, which uses a 6-stage MViT with hierarchical pooling, multi-head attention, and feed-forward networks to classify each proposal. Final action labels are predicted via a linear classifier with softmax activation, outputting probabilities over 8 basketball-specific maneuver classes. BPAN is trained for 60 epochs using the AdamW optimizer with a learning rate of $1 \times 10^{-4}$, batch size 4, and a weight decay of 0.01. Proposal encodings are refined using focal loss (for classification) and generalized IoU loss (for bounding box regression)^78^. To enhance temporal stability, overlapping action proposals are aggregated using confidence-weighted averaging.

At inference time, the BPTN outputs player trajectories and encodings, which are propagated to the BPAN for spatial conditioning of action recognition. Frame-level proposals are generated using sliding windows with a 75% overlap and 25% stride. The final outputs consist of bounding boxes, identity-aware trajectory encodings, and maneuver class predictions. The complete GameSense framework operates at approximately 18 FPS on 720P basketball video, enabling near real-time processing for analytics applications^3,19^. All transformer modules are initialized using Xavier uniform initialization. LayerNorm and dropout (rate = 0.1) are applied to prevent overfitting. Hyperparameters, including memory lengths ($T_s, T_l$), number of proposals, and network depth, were determined through systematic grid search and validated using a held-out portion of the training data.

### Ablation study

#### Ablation study on tracking short-term memory length in BPTN

Figure 6 illustrates the performance of the BPTN with varying short-term memory lengths (1 to 11), keeping the long-term memory length fixed at 28. The results show consistent improvement in metrics as short-term memory length increases, emphasizing the importance of broader context for tracking and identification. At a memory length of 1, BPTN achieves a HOTA of 71.8, IDF1 of 72.2, AssA of 71.4, and MOTA of 81.4, reflecting limited ability to capture complex movements. Performance improves notably at lengths of 3 and 5, with HOTA reaching 78.0 and 82.1, respectively, and MOTA rising from 91.0 to 93.9. The most significant gains occur at a memory length of 7, where BPTN achieves a HOTA of 81.6, IDF1 of 83.1, AssA of 75.0, and MOTA of 97.1, highlighting optimal handling of complex player interactions and long-term dependencies. For lengths of 9 and 11, performance remains robust but shows diminishing returns, with HOTA stabilizing at 79.2 and 80.3, and MOTA at 92.5 and 90.1. A short-term memory length of 7 balances context and computational efficiency, delivering the best overall tracking performance.

**Fig. 7:**
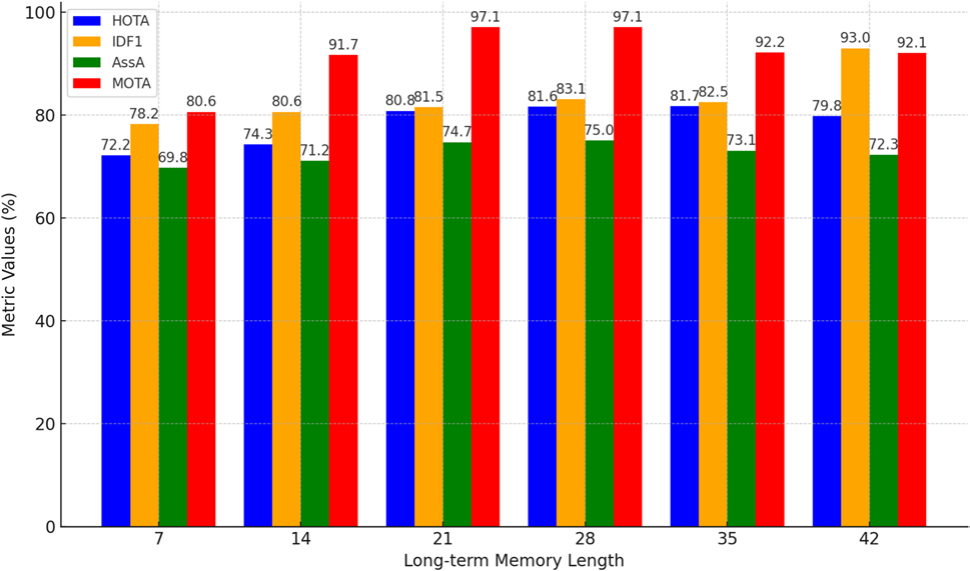
Player tracking performance across varying long-term memory lengths in the BPTN on the SportsMOT dataset, with the short-term memory length fixed at 7.

#### Ablation study on tracking long-term memory length in BPTN

Figure 7 evaluates BPTN’s performance with varying long-term memory lengths, keeping the short-term memory fixed at 7. HOTA consistently improves from 72.2 at a memory length of 7 to a peak of 81.6 at 28, reflecting enhanced detection, association, and localization accuracy. Beyond 28, HOTA stabilizes, with marginal gains (81.7 at 35) followed by a decline (79.8 at 42), suggesting that excessively long memory windows introduce redundant or irrelevant information. Similarly, IDF1 improves from 78.2 at 7 to 93.0 at 42, highlighting reduced identity switches with increased memory. However, metrics such as AssA and MOTA exhibit diminishing returns beyond 28. AssA rises from 69.8 at 7 to 75.0 at 28 but declines to 72.3 at 42. MOTA peaks at 97.1 for memory lengths of 21 and 28 before dropping to 92.1 at 42. The results identify 28 as the optimal long-term memory length, achieving HOTA of 81.6, IDF1 of 83.1, AssA of 75.0, and MOTA of 97.1. These improvements (HOTA +9.4%, IDF1 +6.3%, AssA +5.2%, MOTA +16.5%) demonstrate the importance of balancing memory length to optimize tracking performance without introducing unnecessary complexity or noise.

**Table 1:** Ablation Study on State Encoder Configuration in BPTN. The performance comparison shows the impact of using short-term memory, long-term memory, and their combination on the SportsMOT dataset.

Memory Configuration	HOTA$\uparrow$	IDF1$\uparrow$	AssA$\uparrow$	MOTA↑
Short-term Only	76.9	78.2	68.5	94.8
Long-term Only	78.1	81.0	70.8	95.3
Short + Long-term (Ours)	**81.6**	**83.1**	**75.0**	**97.1**

#### Impact of state encoder configuration in BPTN

To evaluate the role of temporal memory modeling in the BPTN, we conduct an ablation study isolating the contributions of short-term and long-term memory components within the Long-Term Context Buffer module. The goal is to quantify the individual and joint impact of each memory stream on tracking robustness, identity consistency, and association accuracy.

We consider three architectural variants of the State Encoder:

- **Short-term only:** The short-term memory module ($M_s$) is enabled to capture immediate frame-to-frame transitions, while the long-term memory module ($M_l$) is disabled. This variant emphasizes fine-grained temporal locality without accounting for extended player trajectories.
- **Long-term only:** The long-term memory module ($M_l$) is enabled to model extended temporal dependencies, while the short-term memory module ($M_s$) is disabled. This configuration emphasizes global temporal coherence but may struggle with rapid, short-term motion changes.
- **Combined memory (Ours):** Both memory streams are jointly active. The hierarchical fusion module integrates short-term and long-term cues to produce enriched trajectory encodings that balance local precision and global consistency.

Table 1 summarizes the results of these configurations on the SportsMOT dataset. The short-term only configuration yields moderate performance (HOTA = 76.9, IDF1 = 78.2), indicating its effectiveness for recent-frame association but limited long-term identity linking. Conversely, the long-term only setup shows stronger identity retention (IDF1 = 81.0), but lower association precision (AssA = 70.8), likely due to coarse temporal granularity. The baseline configuration achieves the best overall performance (HOTA = 81.6, IDF1 = 83.1, AssA = 75.0, MOTA = 97.1), validating that fusing short- and long-range temporal contexts enables robust, identity-preserving player tracking across dynamic basketball gameplay. These findings highlight the complementary nature of short-term and long-term memory streams in BPTN.

**Fig. 8:**
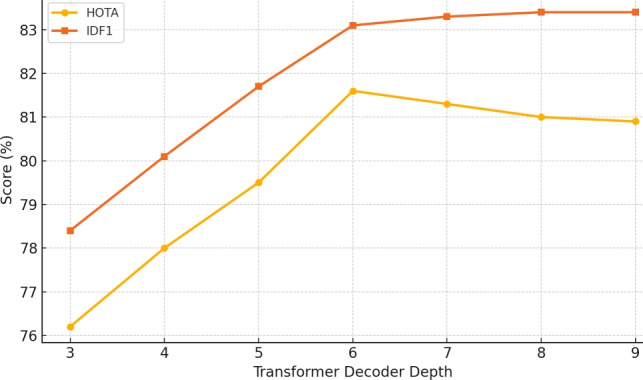
Ablation study showing the effect of Transformer decoder depth on tracking performance in BPTN, evaluated on the SportsMOT dataset. Decoder depth of 6 achieves optimal HOTA and IDF1 trade-off.

#### Ablation study on transformer decoder depth in BPTN

To assess the influence of architectural complexity on tracking performance, we conduct an ablation study on the number of Transformer decoder layers in the BPTN. Specifically, we vary the number of decoder layers from 3 to 9, while maintaining a constant hidden dimension ($d = 256$) and number of attention heads (8) across all configurations. The objective is to analyze how deeper decoder stacks affect the network’s ability to model long-range dependencies, refine trajectory encodings, and maintain identity consistency in dynamic basketball scenarios.

Figure 8 summarizes the tracking performance on the SportsMOT dataset across different decoder depths. At a shallow depth of 3 layers, BPTN achieves a HOTA score of 76.2 and IDF1 of 78.4, indicating limited temporal modeling capacity for complex motion patterns. Increasing the depth to 6 layers yields significant improvements, with HOTA rising to 81.6 and IDF1 to 83.1, demonstrating enhanced capability for contextual alignment and robust player association across frames. However, further increasing the depth to 9 layers leads to marginal gains in IDF1 (83.4) but results in a slight decline in HOTA (80.9), likely due to overfitting and increased computational overhead without proportional benefit. These findings suggest that a decoder depth of 6 offers the best trade-off between model expressiveness and inference efficiency. It effectively captures both local and global temporal cues necessary for accurate player identity tracking, while maintaining tractable computational demands suitable for real-time analytics.

**Table 2:** Ablation study on the choice of Vision Transformer backbone in BPAN. Results are reported on the Basketball-51 dataset.

Backbone	Acc. (%)	Precision (%)	Recall (%)	F1-score (%)
TimeSformer	87.81	86.23	85.72	85.97
Video Swin	89.65	89.01	88.42	88.71
MViT (Ours)	**92.76**	**92.06**	**91.75**	**91.74**

**Fig. 9:**
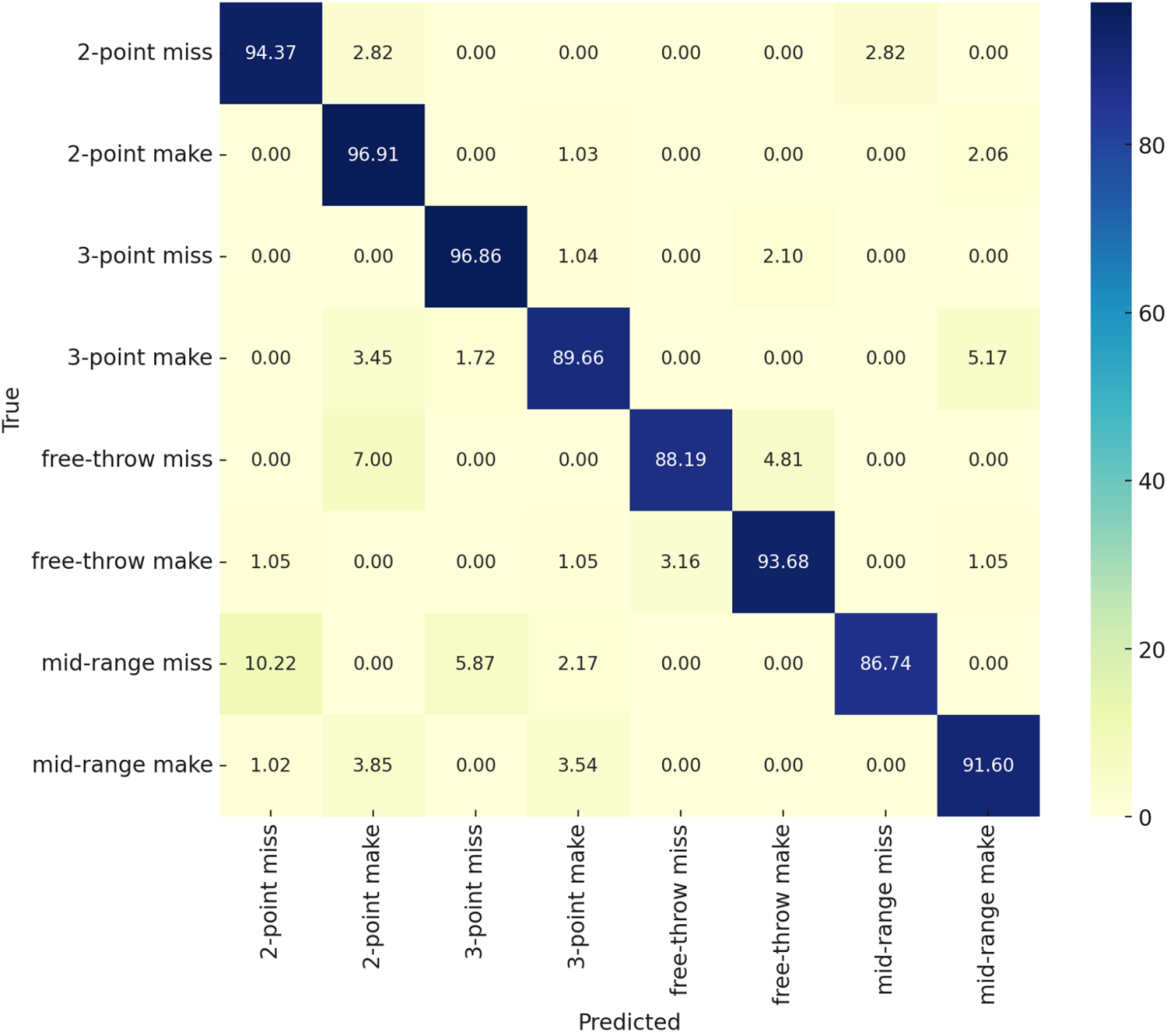
Confusion Matrix of BPAN for basketball action recognition on the Basketball-51 dataset.

#### Impact of vision transformer architecture in BPAN

To evaluate the efficacy of the Multi-Scale Vision Transformer (MViT) as the backbone of the BPAN, we conduct an ablation study comparing it against other prominent Vision Transformer-based architectures: TimeSformer and Video Swin Transformer. Each model was integrated into the BPAN framework and fine-tuned under identical experimental settings on the Basketball-51 dataset.

As shown in Table 2, the MViT backbone demonstrates superior performance across all evaluation metrics. Specifically, it achieves the highest accuracy of 92.76%, surpassing both TimeSformer and Video Swin Transformer, which record 87.81% and 89.65%, respectively. Similarly, MViT achieves the highest F1-score (91.74%) and outperforms its counterparts in both precision (92.06%) and recall (91.75%). These results confirm that MViT’s hierarchical feature learning and multi-scale temporal aggregation provide a more effective representation of basketball-specific motion patterns compared to its single-scale or less temporally expressive alternatives. The improved recognition of subtle maneuver variations and temporal dependencies in fast-paced or partially occluded scenarios, demonstrates that MViT is better equipped to capture the complex spatio-temporal dynamics inherent in basketball gameplay. This justifies its selection as the preferred backbone for the BPAN framework.

#### Class-wise performance analysis in BPAN

The confusion matrix in Fig. 9 illustrates BPAN’s performance for basketball action recognition across eight action classes in the Basketball-51 dataset. Diagonal elements indicate correct classifications, while off-diagonal elements show misclassifications. High recognition accuracies are observed for actions like *2-point miss* (94.37%), *2-point make* (96.91%), and *3-point miss* (96.86%), reflecting BPAN’s ability to capture distinct spatial-temporal patterns. Similarly, *free-throw miss* and *free-throw make* achieve 88.19% and 93.68% accuracy, respectively, due to the static and uniform context of these actions.

Lower accuracies for *mid-range miss* (86.74%) and *mid-range make* (91.60%) indicate challenges in distinguishing overlapping features with other classes like *2-point* and *3-point* actions. Misclassifications, particularly between *mid-range* and *free-throw* actions, are likely caused by shared spatial contexts and similar shot mechanics. BPAN’s strong performance on distinct actions highlights its effectiveness in modeling dynamic and static scenarios, leveraging spatio-temporal transformer architecture. However, improved differentiation for overlapping classes may require enhanced contextual features, such as ball trajectory or court zones, and a more diverse dataset to improve generalization and reduce ambiguity.

**Table 3:** Ablation study on the number of proposal queries in the Action Proposal Module.

Queries	Recall (%)	Proposal Precision (%)	Action mAP (%)
5	84.7	85.3	86.1
**10 (Ous)**	**89.2**	**88.5**	**91.7**
15	89.8	87.2	90.9

**Table 4:** Ablation study on temporal stride and clip length in BPAN. Performance is reported on the Basketball-51 dataset.

Clip Length	Stride	Accuracy (%)	F1-score (%)
32	2	89.12	87.90
32	4	88.45	87.13
32	6	86.30	84.72
64	2	91.34	90.51
**64 (ours)**	**4 (ours)**	**92.76**	**91.74**
64	6	91.02	89.68
96	2	90.12	89.41
96	4	90.01	88.92
96	6	88.73	87.06

#### Impact of proposal query count in BPAN

This ablation investigates the influence of varying the number of proposal queries within the Action Proposal Module on the effectiveness of action segmentation and classification. Proposal queries serve as learnable encodings that attend to spatio-temporal feature maps to localize potential action segments. Adjusting their count directly affects the granularity and diversity of generated action proposals. We evaluate three configurations: 5, 10 (baseline), and 15 proposal queries on the Basketball-51 dataset.

As summarized in Table 3, using 5 queries results in limited proposal diversity and reduced action coverage, achieving an action mAP of 86.1% and Recall of 84.7%. Increasing the count to 10 enhances proposal granularity, leading to improved action mAP (91.7%) and Recall (89.2%), while maintaining high Proposal Precision (88.5%). Further increasing the query count to 15 yields marginal gains in Recall (89.8%) but results in a slight drop in precision (87.2%) due to increased proposal redundancy and overlapping segments, which may introduce false positives in classification. These findings indicate that 10 proposal queries provide an optimal trade-off between coverage and precision, capturing sufficient action diversity without over-segmenting or overfitting. Therefore, the default configuration in BPAN uses 10 queries to balance computational cost and proposal quality.

#### Ablation study on temporal stride and clip length

To evaluate the impact of temporal sampling strategies on the performance of the BPAN, we conduct a comprehensive ablation study by varying the temporal stride and input clip length. Temporal stride controls the interval at which frames are sampled from the raw video, while clip length determines the number of frames used to form each proposal clip. These two parameters directly influence the temporal resolution and contextual span of the input to the action recognition model. We consider clip lengths of 32, 64 (baseline), and 96 frames, each sampled at temporal strides of 2, 4, and 6. All configurations are evaluated on the Basketball-51 dataset using classification **accuracy** and **F1-score** as the primary performance metrics. The results are summarized in Table 4.

From the results, we observe that shorter clip lengths (e.g., 32 frames) lead to performance degradation, particularly at higher strides, due to the insufficient temporal context for modeling complex maneuvers. Conversely, extremely long clips (96 frames) sampled with low strides incur redundancy and noise, diminishing the model’s discriminative capacity. The best overall performance is achieved with a clip length of 64 frames and a stride of 4, yielding an accuracy of 92.76% and an F1-score of 91.74%. This configuration provides a balanced trade-off between temporal resolution and contextual coverage, effectively capturing both rapid transitions and sustained player movements.

**Table 5:** Performance Comparison with recent trackers on SportsMOT. A dash indicates the metric was not reported by the original paper.

Model	HOTA$\uparrow$	IDF1$\uparrow$	AssA$\uparrow$	MOTA$\uparrow$	DetA$\uparrow$	IDs$\downarrow$
FairMOT^16^	49.3	53.5	34.7	86.4	70.2	9928
QDTrack^21^	60.4	62.3	47.2	90.1	77.5	6377
BoT-SORT^23^	68.7	70.0	55.9	94.5	84.4	6729
ByteTrack^20^	64.1	71.4	52.3	95.9	78.5	3089
OC-SORT^18^	73.7	74.0	61.5	96.5	88.5	2728
MixSort-OC^19^	74.1	74.4	62.0	96.5	88.5	2781
Deep-EIoU^79^	77.2	79.8	67.7	96.3	88.2	2659
DiffMOT^80^	76.2	76.1	65.1	97.1	87.7	–
SportMamba^81^	77.3	77.7	66.8	96.9	87.5	–
McByte^82^	76.9	77.5	–	**97.2**	–	–
**BPTN (ours)**	**81.6**	**83.1**	**75.0**	97.1	**88.3**	**2164**

### Performance comparison with SoTA models

#### Player tracking performance comparison

Table 5 reports a comprehensive comparison on SportsMOT across classic and recent sports-oriented trackers, including 2025 entries (SportMamba and McByte) and an updated DiffMOT line with full metrics. The proposed BPTN attains the highest HOTA (81.6) among all methods, exceeding strong sports baselines such as Deep-EIoU ^79^ (77.2), DiffMOT ^80^ (76.2), SportMamba ^81^ (77.3), and McByte ^82^ (76.9). In terms of identity preservation, BPTN also achieves the top IDF1 (83.1), comfortably outperforming the next best recent entries (SportMamba 77.7; McByte 77.5; DiffMOT 76.1). Association quality follows the same trend, with BPTN delivering the best AssA (75.0) versus Deep-EIoU (67.7), SportMamba (66.8), and DiffMOT (65.1), indicating more reliable long-range identity continuity under occlusion and non-linear player motion.

For MOTA, BPTN (97.1) is competitive with the strongest detectors and association stacks, matching DiffMOT (97.1) and trailing McByte’s best (97.2) by a negligible margin. On detection accuracy, BPTN’s DetA (88.3) is competitive with high-performing tracking-by-detection baselines (e.g., OC-SORT/MixSort-OC at 88.5), while delivering substantially stronger association quality overall. Importantly, BPTN records the lowest number of identity switches (IDs = 2164), improving upon sports-focused Deep-EIoU (2659) and classical baselines alike. These results show that BPTN’s memory-conditioned decoding and dual-scale temporal context yeld SoTA holistic tracking performance on SportsMOT achieving the best balance of detection-association integration and identity stability.

**Table 6:** Performance comparison of our BPAN with SoTA models on the Basketball-51 dataset for the action recognition task.

Model	Accuracy	Precision	Recall	F1-score
TSM^78^	81.21	83.90	77.08	79.68
ViViT^11^	84.88	67.13	65.77	66.31
Video-Swin^10^	85.08	85.23	80.51	81.22
ACA-Net^83^	92.05	90.44	89.39	89.68
BPAN (Ours)	**92.76**	**92.06**	**91.75**	**91.74**

#### Action recognition performance comparison

The results presented in Table 6 provide a comprehensive performance comparison of the proposed BPAN against SoTA models on the Basketball-51 dataset for the action recognition task. Proposed BPAN outperforms all existing SoTA models across all metrics. Specifically, BPAN achieves an accuracy of 92.76%, surpassing ACA-Net^83^ (92.05%) and significantly outperforming Video-Swin^10^ (85.08%),^11^ (84.88%), and^78^ (81.21%). This substantial improvement in accuracy highlights BPAN’s superior ability to correctly classify basketball actions compared to these advanced models.

In terms of precision, BPAN again leads with a score of 92.06%, outperforming ACA-Net (90.44%) and Video-Swin (85.23%). Precision is crucial in action recognition as it reflects the model’s ability to avoid false positives. BPAN’s high precision demonstrates its capacity to identify the correct actions with minimal misclassification. For recall, BPAN achieves 91.75%, surpassing ACA-Net (89.39%) and Video-Swin (80.51%). Recall measures the model’s ability to identify all relevant actions, and BPAN’s high score emphasizes its effectiveness in capturing most basketball actions from video data, ensuring few actions are missed. Finally, BPAN records an F1-score of 91.74%, again outperforming ACA-Net 89.68%) and Video-Swin (81.22%). The high F1-score demonstrates BPAN’s balanced trade-off between precision and recall, ensuring robust action recognition.

### Discussion

The proposed integrated framework, comprising the BPTN and BPAN, demonstrates superior performance, significantly surpassing existing state-of-the-art methods. This discussion explicitly analyzes the distinctive technical aspects and contributions responsible for the superior outcomes, supported by detailed ablation studies and comparative evaluations.

#### Distinctive features of BPTN

The BPTN’s performance improvements arise from several unique design enhancements. BPTN incorporates dual-level hierarchical temporal memory, consisting of distinct short-term and long-term memory modules. Unlike conventional single-scale temporal modeling, this hierarchical structure adeptly captures both immediate player movements and extended trajectory contexts. Ablation studies explicitly validate that individually using short-term or long-term memory achieves lower performance metrics, while their combination significantly boosts overall performance (HOTA: 81.6, IDF1: 83.1). Specifically, short-term memory (optimal length of 7 frames) captures fine-grained, rapid positional changes, whereas long-term memory (optimal length of 28 frames) robustly preserves identity continuity over prolonged intervals.

Unlike traditional tracking methods that independently handle object detection and identity association, BPTN integrates a memory-driven transformer decoder. The ablation study on transformer decoder depth explicitly demonstrates that a decoder depth of six layers provides optimal context modeling capability, balancing computational complexity and performance accuracy (HOTA of 81.6 at depth 6 compared to lower scores at shallower depths). This decoding approach ensures superior alignment of spatial detections with temporal encodings, effectively minimizing identity switches, as evidenced by the lowest IDs metric (2164 compared to 2659 in Deep-EIoU).

#### Distinctive features of BPAN

The exceptional action recognition performance of BPAN results from distinct architectural choices. BPAN employs an advanced multi-scale vision transformer architecture explicitly capturing complex spatio-temporal dynamics at multiple scales. Ablation studies demonstrate MViT’s clear superiority over alternative vision transformer architectures like TimeSformer and Video-Swin, yielding the highest action recognition metrics (accuracy: 92.76%, precision: 92.06%). This advantage arises from MViT’s hierarchical pooling attention, efficiently aggregating both local and global contextual features essential for accurately differentiating subtle maneuver variations, particularly critical in basketball scenarios. BPAN incorporates an explicit action proposal module using transformer-based encoding and decoding to localize action segments accurately. Ablation studies regarding the optimal number of proposal queries explicitly reveal that using ten proposal queries achieves the ideal trade-off between capturing action diversity and maintaining high precision (Action mAP: 91.7%). Fewer proposals reduce recall, while excessive proposals introduce redundancy and diminish precision.

BPAN adopts a carefully optimized temporal sampling strategy, selecting clips of 64 frames with a stride of 4 frames, proven optimal through rigorous ablation. Shorter clips fail to provide adequate temporal context, while longer clips introduce redundancy and noise, explicitly affecting performance negatively. The selected temporal parameters explicitly balance temporal granularity and computational efficiency, leading to robust recognition accuracy (F1-score: 91.74%). Despite evident strengths, some limitations remain. Specifically, moderate difficulty distinguishing certain mid-range actions was identified, highlighting areas for potential improvement. Explicitly integrating additional contextual cues such as ball trajectory, court geometry, and strategic contexts may mitigate these challenges in future developments.

### Computational cost and scalability analysis

GameSense framework is designed with scalability and efficiency in mind to support real-time analytics in competitive basketball environments. From a computational perspective, the BPTN module operates at $\sim$18 FPS on 720p video using a single NVIDIA RTX 3090 GPU, enabled by efficient detection via YOLOv7 and a 6-layer transformer decoder stack with moderate hidden dimensions (256). The memory buffer design, with fixed short-term ($T_s=7$) and long-term ($T_l=28$) windows, ensures that temporal modeling remains bounded in both time and memory complexity, avoiding unbounded temporal growth as game duration increases.

BPAN leverages a modular sliding-window mechanism with 64-frame clips sampled at a stride of 4, allowing for adjustable temporal granularity while maintaining throughput. The use of a 6-stage MViT, instead of deeper architectures like ViViT or I3D, achieves a favorable trade-off between temporal expressiveness and latency. Action proposals are limited to 10 learnable queries, minimizing redundant computation. The overall system supports batch inference and parallel GPU acceleration for both tracking and recognition stages.

In terms of scalability, GameSense is deployable on commodity hardware and supports online inference. Its modularity allows decoupling the tracking and recognition components for distributed deployment (e.g., edge detection + cloud analytics). The model is trained on large-scale, diverse datasets (SportsMOT, Basketball-51), and its architecture generalizes to other team sports with similar player dynamics. These properties collectively ensure the framework’s computational tractability and deployment feasibility in real-world sports analytics scenarios.

#### Failure case analysis

While the proposed GameSense framework demonstrates strong tracking and action recognition capabilities in dynamic basketball environments, several failure modes were observed that reflect the inherent challenges of real-world sports analytics. These cases highlight opportunities for further refinement.

**Identity switches in overlapping scenarios** In dense gameplay situations, especially during screen plays or fast breaks, multiple players often converge spatially, leading to overlapping bounding boxes. Although the memory-driven decoder in BPTN incorporates both visibility and distinctiveness scores for identity association, ambiguity in spatial proximity can result in identity switches. This occurs when distinctiveness cues are weak (e.g., players on the same team with similar appearance) and the decoder cannot confidently resolve player identities.

**Track fragmentation after occlusion** Prolonged occlusion, such as a player being fully blocked by another or a referee, occasionally disrupts track continuity. When the occlusion exceeds the long-term memory window ($T_l = 28$), the association mechanism fails to recover prior identity. This leads to the premature termination of existing tracks and initialization of new identities upon reappearance. Although the hierarchical memory mitigates short-term disruptions, recovery under extended occlusion remains limited.

**Bounding box drift during camera panning** Rapid camera transitions or zooming can introduce detection jitter, causing bounding boxes to shift unpredictably across consecutive frames. These distortions propagate into the memory buffer, degrading the temporal consistency of embeddings and leading to suboptimal attention weight assignments in the decoder. This is particularly problematic during transitions between half-court and full-court views, where the background and scale of player representations change abruptly.

**Ambiguity in similar Maneuver classification** Despite BPAN’s use of MViT for robust temporal modeling, misclassification occurs between visually and temporally similar actions such as *mid-range shots* vs. *free throws*, or *three-point attempts* vs. *long-range passes*. These classes often share body posture and release mechanics, making them difficult to disambiguate without contextual cues like ball trajectory or court markings. Limited spatial resolution or partial occlusion further exacerbate these misclassifications.

**Under-segmentation of fast actions** BPAN relies on overlapping 64-frame clips for action segmentation. In fast-paced sequences where multiple short actions occur in succession (e.g., rebound followed by put-back shot), the temporal window may group multiple actions into a single proposal. This under-segmentation leads to diluted temporal attention, causing misalignment between proposal boundaries and actual maneuver start-end points.

**Jersey number confusion in Re-identification** When players from the same team wear similar uniforms and occlusion obscures jersey numbers or distinguishing features, track associations rely heavily on motion continuity. In cases of sudden stops or directional changes, motion priors fail, and players may be incorrectly re-identified. This highlights the need for integrating fine-grained appearance cues (e.g., pose or number recognition) in ambiguous re-identification scenarios.

**Low-light and shadow artifacts** In poorly lit indoor arenas or under harsh lighting conditions, shadows and low-contrast regions can degrade detection performance. YOLOv7 occasionally produces false positives (e.g., shadows cast on the floor) or misses detections in dim regions. These detection anomalies cascade into the tracking and recognition pipeline, resulting in false trajectories and dropped action segments.

**Three-point vs. long two at Arc boundary** One failure mode involves the misclassification of three-point shots versus long two-point attempts when the shooter’s feet are positioned near the arc boundary. This ambiguity primarily arises at a 720p resolution, where downsampling diminishes the spatial separability between the player’s foot position and the arc line in the encoded patch tokens. Consequently, the Multi-Scale Vision Transformer (MViT) struggles to discern whether the player is positioned just inside or beyond the three-point line leading to inconsistent maneuver classification. To mitigate this issue, we propose a line-aware super-resolution crop centered on the shooter’s feet, coupled with a binary auxiliary classifier that leverages a homography-aligned arc mask to determine whether the shot originates beyond the arc. This output can be fused as a logit-space calibration before refining the primary MViT-based classification and improving the decision boundary in such fine-grained spatial scenarios.

**Mid-range vs. Free-throw confusions** Another frequent misclassification occurs between mid-range shots and free-throw attempts, particularly when stationary shooters are positioned near the elbow. These instances are often incorrectly labeled as free throws (*ft1/ft0*) instead of the correct mid-range class (*mp1/mp0*). The primary cause stems from the lack of court semantics, such as the free-throw semicircle or key within the spatial extent of the proposal crops, as well as the absence of explicit ball trajectory cues. Moreover, the temporal context in such clips is typically static, offering insufficient motion dynamics to disambiguate the two actions. To address this, we propose injecting court-zone priors via single-view homography transformations that map the shooter’s position relative to canonical court zones. In addition, a lightweight ball-trajectory prediction head can provide auxiliary cues about shot type, while extending the proposal context by ±8 frames for low-motion sequences introduces additional temporal information to differentiate between structured free-throw routines and more dynamic mid-range plays.

**Transition plays with camera shake** A critical failure mode emerges during transition plays, especially fast breaks involving long passes and rapid camera movements. These instances often result in fragmented action proposals and misclassification of layups due to the temporal disconnection between the pass and shot events. The root cause lies in pan and zoom-induced motion blur, which violates the model’s assumed stride-4 temporal consistency, leading to frame-to-frame misalignment across the 64-frame proposal windows. Consequently, high-motion segments are poorly represented, and the action boundaries become ambiguous. To mitigate this, we propose applying per-clip affine stabilization to reduce camera-induced distortion, alongside using an adaptive temporal stride lowering it to $S=2$ in high-motion intervals to capture finer temporal granularity. Furthermore, we introduce hysteresis-based merging for temporally overlapping proposals, allowing smoother aggregation of fragmented clips into coherent action sequences and improving recognition of complex multi-step transitions.

## Conclusion

This study introduces a comprehensive framework for basketball analytics, integrating the Basketball Player Tracking and Analytics Network to address challenges in player tracking and action recognition. The BPTN employs a hierarchical temporal memory mechanism and transformer-based architecture to enhance tracking precision, association accuracy, and identity consistency. Its Long-Term Context Buffer module effectively balances short-term and long-term dependencies, ensuring robust player identity maintenance under complex conditions like occlusions and overlapping trajectories. For action recognition, BPAN leverages multi-scale vision transformers with hierarchical temporal processing, achieving accurate classification of basketball maneuvers while handling temporal and spatial complexities. The modular action proposal and recognition design ensure efficient segmentation and classification, delivering state-of-the-art results on benchmark datasets. Beyond tracking and action recognition, the framework integrates contextual and temporal information, providing insights into gameplay dynamics such as team coordination, player roles, and strategic maneuvers. Future work can focus on improving the model’s differentiation of similar actions, such as mid-range and free-throw shots, by incorporating contextual features like ball trajectory and court zones. Extending the framework to multi-camera setups and diverse sports domains could further enhance its applicability and scalability.

## Data Availability

The datasets used in the experiments are publicly available. The SportsMOT dataset is available accessed at https://datasetninja.com/sports-mot, and the Basketball-51 dataset is available at https://www.kaggle.com/datasets/sarbagyashakya/basketball-51-dataset.
